# Translation and measurement properties of the Portuguese-Brazil version of the Hammersmith Infant Neurological Examination (HINE-Br)

**DOI:** 10.1590/1984-0462/2024/42/2023105

**Published:** 2024-03-25

**Authors:** Michelle Alexandrina dos Santos Furtado, Hércules Ribeiro Leite, Matheus Rocha Pereira Klettenberg, Victor Alves Rodrigues, Lisiane Seguti Ferreira, Melina Rodero Marques, Isadora de Oliveira Cavalcante, Tamires Saboia Santos, Tathiana Ghisi de Souza, Ayrles Silva Gonçalves Barbosa Mendonça, Ana Cristina Resende Camargos, Kênnea Martins Almeida Ayupe

**Affiliations:** a Universidade Federal de Minas Gerais (UFMG), Belo Horizonte, Minas Gerais, Brazil.; b Universidade de Brasília (UnB), Brasília, Distrito Federal, Brazil.; c Faculdade de Medicina de Jundiaí (FMJ), Jundiaí, São Paulo, Brazil.; d Universidade Federal do Amazonas (UFAM), Manaus, Amazonas, Brazil.; e Universidade Federal de Minas Gerais (UFMG), Belo Horizonte, Minas Gerais, Brazil.

**Keywords:** Early diagnosis, Neurological examination, Infant, Cerebral palsy, Translations, Reproducibility of tests, Diagnóstico precoce, Exame neurológico, Lactente, Paralisia cerebral, Traduções, Reprodutibilidade dos testes

## Abstract

**Objective:**

The current study aimed to translate the Hammersmith Infant Neurological Examination (HINE) into Brazilian Portuguese and analyze the reliability of the translated version for a population of Brazilian infants.

**Methods:**

This was a methodological study, approved by the Ethics Committee, carried out between June 2020 and May 2021. HINE is a standardized clinical neurological examination used for the early detection of cerebral palsy. The quantitative section, “neurological examination”, contains 26 items scored from 0 to 3 points, divided into five categories: cranial nerve function, posture, movements, muscle tone and reflexes, and reactions. The HINE translation followed four steps: translation, synthesis, back-translation, and evaluation by an expert committee. To verify the reliability of the HINE-Br (Portuguese-Brazil version) two independent examiners evaluated 43 infants, between 3 and 22 months of age. Internal consistency was verified by Cronbach’s Alpha coefficient and interrater reliability by the intraclass correlation coefficient (ICC).

**Results:**

The translated version was similar to the original version and a few semantic and idiomatic adjustments were necessary. Appropriate internal consistency (Alpha=0.91) was found for the 26 items of the HINE-Br, as well as strong interrater reliability for the total score (ICC_2.1_=0.95), and also for the five categories (ICC_2.1_=0.83–0.95).

**Conclusions:**

The HINE-Br presents adequate rates of internal consistency and interrater reliability, and can be used for the evaluation of children at risk for cerebral palsy, between 3 and 24 months of age, by pediatricians and pediatric physical therapists.

## RESUMO

**Objetivo:**

Traduzir o *Hammersmith Infant Neurological Examination* (HINE) para o português brasileiro e analisar a confiabilidade da versão traduzida em lactentes brasileiros.

**Métodos:**

Estudo metodológico, aprovado por Comitê de Ética, realizado entre junho de 2020 e maio de 2021. O HINE é um exame clínico neurológico padronizado, utilizado para detecção precoce de paralisia cerebral. A seção quantitativa, “exame neurológico”, contém 26 itens pontuados de 0 a 3, divididos em 5 categorias: função dos nervos cranianos; postura; movimentos; tônus muscular e reflexos; e reações. A tradução do HINE seguiu quatro etapas: tradução; síntese; retrotradução; e avaliação por um comitê de especialistas. Dois examinadores independentes avaliaram 43 lactentes, entre 3 e 22 meses, utilizando a versão HINE-Br (versão em português brasileiro), para verificar sua confiabilidade. A consistência interna foi verificada pelo coeficiente Alpha de Cronbach e a confiabilidade interexaminadores pelo coeficiente de correlação intraclasse (CCI).

**Resultados:**

A versão traduzida foi semelhante à versão original e poucos ajustes semânticos e idiomáticos foram necessários. Encontrou-se consistência interna adequada (Apha=0,91) para os 26 itens do HINE-Br, bem como forte confiabilidade interexaminadores para o escore total (CCI_2,1_=0,95) e também para as cinco categorias (CCI_2,1_=0,83–0,95).

**Conclusões:**

O HINE-Br apresenta índices adequados de consistência interna e confiabilidade interexaminadores, podendo ser utilizada para avaliação de crianças com risco de apresentar paralisia cerebral, entre 3 e 24 meses de idade, por pediatras e fisioterapeutas infantis.

## INTRODUCTION

Cerebral palsy (CP) is characterized by a disorder of posture and movement development, caused by malformations or by damage in the immature brain (fetal or infant).^1^ The global prevalence in developed countries is about 1.6 per 1000 live births; however, it is estimated that these rates are higher in Low- and Middle-Income Countries (LMICs).^2^ CP is the main form of physical disability in childhood, with important mobility limitations and participation restrictions.^3^ Clinical subtypes of CP are classified according to the primary motor disorder manifestation, such as spastic, dyskinetic, or ataxic, and may present bilateral or unilateral involvement.^1^ Children with CP also present intellectual disability (49%), epilepsy (35%), secondary musculoskeletal impairments (28%), behavioral disorders (26%), visual disorders (11%), and hearing impairment (4%), among other complications.^1,4^

The main causes of CP are related to pre, peri, and postnatal risk factors, such as prematurity, low birth weight, hypoxic-ischemic encephalopathy, respiratory distress syndrome, neonatal seizures, neonatal intracranial hemorrhage, and infections, among others.^5^ In LMICs, risk factors may also be related to low assistance from health services in pre, peri, and postnatal periods, socioeconomic status, family education level, and limited access to specialized health care.^6,7^

The diagnosis of CP is clinical, and is usually made after two years of age, which makes it difficult to manage some disabilities that could be prevented with early interventions.^8^ Otherwise, the early detection of infants with a “high risk of CP” allows the implementation of effective therapeutic strategies in the prevention and treatment of some disabilities.^4,8^ According to the International Clinical Guideline for Early Detection of CP,^4^ children at a “high risk of CP” can be accurately identified earlier using three main exams: Neonatal Nuclear Magnetic Resonance (NMR), Prechtl Qualitative Assessment of General Movements (GMs), and the Hammersmith Infant Neurological Examination (HINE).^4,8^ The literature shows that high-income countries which incorporate contemporary guidelines for early detection and intervention, present a decrease in the mean age at diagnosis of CP compared to LMICs.^9,10^

Among the above mentioned three main exams, the HINE is the most feasible tool for the early detection of CP in clinical practice, as it is easy to apply and presents a low cost, making it very useful in public services.^11^ The HINE has good predictive criterion validity (90%), interrater reliability (≅100%), high sensitivity (90%), and specificity (85%) for CP detection from 2 to 24 months of corrected age.^4,12^ In addition, the HINE can provide information on neuromotor development at 3 to 6 months of age, and relate gross motor functional skills at 2 years of age to levels on the Gross Motor Function Classification System (GMFCS).^4,13^

Thus, there is a lack of published literature on surveillance and early detection implementations by health professionals in the follow-up of high-risk CP babies in LMICs.^10^ In addition, LMICs, like Brazil, present uncertain epidemiological data on Brazilian children with CP and the use of standardized clinical tools in the diagnosis of this health condition is still poorly studied.^14-16^ One of the tools used to early diagnose CP in LMICs specifically is the NMR; however it is not accessible to the majority of the population, and there is a lack of information on how CP diagnosis is performed in the clinical practice of Brazilian services. The absence of standardized and valid tools available in Portuguese and adapted for the Brazilian population with CP, as well as the absence of Brazilian guidelines, may contribute to the failure of early detection of CP in the country and the lack of epidemiological data. Recently, the translation of one of the versions of the HINE, the Hammersmith Neonatal Neurological Examination (HNNE), was published, which is intended only for the assessment of newborns up to the first month of age.^14^ Consequently, the translation of the HINE into Brazilian Portuguese will allow the expansion of early detection of CP in infants up to 24 months of age. Thus, the objectives of the current study were:

1. To translate the HINE into Brazilian Portuguese (HINE-Br) and
2. To analyze the reliability of the HINE-Br in the population of Brazilian infants.

## METHOD

This methodological study was based on the taxonomy, terminology, and definitions of measurement properties of the Consensus-based Standards for the selection of health status Measurement Instruments (COSMIN).^17^

The study was divided into two stages:

1. Translation from English into Brazilian Portuguese and
2. Reliability analysis (internal consistency and interrater reliability).

The study was conducted at the University Hospital of Brasília (HUB), Brazil, between June 2020 and May 2021, and was approved by the Ethics and Research Committee (CAAE: 30766620.1.0000.8093).

### Participants

Infants between 3 and 24 months of age or corrected age (in the case of preterm infants) were selected from an outpatient follow-up of children at a high risk for developmental disability in HUB. Infants with a history of prematurity, neonatal complications, and motor delay were included. Infants who cried or were not alert during the assessment were excluded. Personal data and clinical history were collected from HUB’s electronic medical records. Parents were invited to participate voluntarily and those who agreed signed the Free and Informed Consent Term.

The HINE is a standardized clinical neurological examination used for the early detection of CP in preterm and term infants between 3 and 24 months of age.^18^ It is simple, quick, easy to access, and free, and can be used by pediatricians, pediatric neurologists, and multidisciplinary teams with experience in the area of child rehabilitation, after familiarization with the instrument.^3^ The HINE Recording & Scoring Proforma (score sheet) contains all the items of the test. There is also a book and a website that provide training videos and a detailed description of the application.^18,19^

The HINE contains three sections. Section 1, the “neurological examination”, is quantitative, containing 26 items scored from 0 to 3 points, and is divided into five categories: cranial nerve function, posture, movements, muscle tone and reflexes, and reactions. The total or global score of this section ranges from 0 to 78 points, and the lower the score the higher the risk of CP. Global scores are reported as optimal if they are equal to or above 73 at 9 to 12 months of age, or equal to or above 70 and 67 at 6 and 3 months, respectively.^20^ Regarding cutoff points, infants who have scores ≤58 at 3 months of age, ≤64 at 6 months, and ≤69 at 9 to 12 months are considered at risk for CP, and infants who score ≤40 from 3 months of age present CP.^4,12^ Sections 2 and 3, namely “motor milestones” and “behavior”, respectively, are qualitative and provide additional information on the interpretation of the test.^21^

The translation process was carried out according to guidelines from Beaton et al.^22^ and Guillemin et al.^23^ The HINE website expert team (Dr. M. M. B.) approved our request to translate the HINE Recording & Scoring Proforma into the Brazilian Portuguese language and monitored the process. The translation involved five independent steps. Step 1: initial translation of the HINE from English to Brazilian Portuguese by three independent translators who had Portuguese as their first language and were fluent in English (two health professionals and one language professional translator). Step 2: consensus of all translators from step 1. Step 3: HINE back-translation from the Brazilian Portuguese language to English by three independent back-translators (two health professionals and one language professional translator). Step 4: consensus of all back-translators from step 3. Step 5: a committee of experts (i.e., researchers and professionals in the field of pediatric neurology) revised and reached a consensus on the pre-final version of the HINE-Br Recording & Scoring Proforma, taking into consideration its original version, the translated version, and the back-translated version.

In step 5, the following possible solutions were explored for the discrepancies between the versions: addition (adding technical terms to clarify the item), word replacement (according to common linguistic expressions among health professionals in Brazil), and sentence organization (to make ideas clearer).^24^ Thus, the pre-final HINE-Br Recording & Scoring Proforma was submitted for careful review by the website’s expert team. After the suggestions provided by the test developers (Dr. M. M. B.) and the last adjustments, the final version was successful and was called the “*Exame neurológico infantil de Hammersmith*”, HINE-Br.^25^

We analyzed the reliability of the HINE-Br by calculating the internal consistency and the interrater reliability. Internal consistency is whether the items of an instrument are inter-correlated (homogeneous), i.e., the items of an instrument measure the same construct. Interrater reliability is the estimate of how consistent the instrument is when applied by different examiners to assess the same subjects.^17^ For reliability studies, the COSMIN^17^ recommends a sample of 50 participants.

Descriptive data of the participants, including sex, age, and neonatal complications, were collected from each infant’s medical record. Four examiners (neuropediatric residents, and physical therapists) with over 5 years of experience in assessments and treatment in the children’s area, were trained in the application of the HINE-Br by reading the manual, watching the training video,^19^ through an online workshop with experts (4 h), and a face-to-face pilot study with five infants aged 3, 6, 9, 12 and 15 months old, showing good reliability rates among them. All assessments were performed when the infants were alert, active, and wearing minimal clothing. The four examiners conducted the assessments in pairs. During the assessment, examiner I applied each HINE item while examiner II observed and independently scored the items on his score sheet. In items that require manipulation (i.e., “tone” items), examiner II performed the examination of the item by himself to better judge the child’s response, without discussion with examiner I. Assessments lasted approximately 20 minutes.

We used descriptive statistics to summarize the characteristics of the participants. For continuous variables, we used mean and standard deviation (SD), and for categorical variables we applied absolute frequencies and percentages. To calculate the internal consistency, we considered the item scores performed by examiner I. Internal consistency was measured using Cronbach’s Alpha coefficient. This coefficient ranges from 0 to 1, where scores between 0.70 and 0.95 are considered appropriate.^26^

To analyze the interrater reliability between the scores of examiners I and II we used the intraclass correlation coefficient (ICC_2.1_), and absolute agreement with two-way random analysis and its respective 95% confidence interval (CI). The ICC value was interpreted as: ≤0.25 very weak correlation; 0.25–0.50 weak; 0.50–0.75 moderate; and >0.75 strong correlation.^26^ All statistical analyses were performed through the Statistical Package for Social Sciences (SPSS) (version 22.0), considering α=0.05.

## RESULTS

We invited 55 infants to participate in this study, of whom 12 were excluded for crying during the exam, which had to be stopped. Finally, 43 infants between 3 and 22 months of age (mean=7.84) participated, and their characteristics are described in Table 1. The most prevalent risk factors for CP among the participants were long hospital stay (61.5%), prematurity (28.2%), respiratory distress (25.6%), extreme/low weight at birth (20.5%), and resuscitation or sepsis (17.9%).

**Table 1:** Characteristics of the participants.

Variables	n (%) or mean ± SD
Months of age	7.8±5.5
Female	25 (58.1)
Evaluation age (months)	
3 to 5	24 (55.8)
6 to 11	9 (20.9)
12 to 24	10 (23.3)
HINE total score	57.7±14.27
CP diagnosis*	7 (16.3)

SD: standard deviation; n (%): absolute number and percentage; HINE: Hammersmith Infant Neurological Examination; CP: cerebral palsy.

*infants who scored ≤40.

The expert committee and the instrument developer identified some discrepancies between the original version and HINE-Br. To resolve these discrepancies, the committee used addition, word replacement, and organization of sentences as strategies to obtain semantic and idiomatic equivalence. Table 2 shows some examples of discrepancies found during the translation process and the strategies used to resolve them. The HINE-Br (Supplementary Material 1) is available on the instrument’s official website https://www.mackeith.co.uk/pt_br/hammersmith-neurological-examinations/hammersmith-neurological-examinations-subscriber-content/.

**Table 2:** Examples of discrepancies found during the translation process and the strategies used by the expert committee group and the Hammersmith Infant Neurological Examination developer.

Item	Description in the original English version	Description in the translated Portuguese-Brazil version	Strategy
Sub section – assessment of cranial nerve function			
Eye movements	Score 3 Normal conjugate* eye movements	Escore 3 Movimentos normais e coordenados (ambos os lados)*	Word replacement/addition
Sub section – assessment of posture			
Feet in supine andin standing	Score 3 Toes straight midway between flexion and extension	Escore 3 Dedos retos entre semi flexão e extensão (em posição neutra)*	Addition
Sub Section – Reflexes and reactions			
Tendon reflexes	Score 2 Mildly brisk bicep knee ankle	Escore 2 Levemente ativo Bíceps joelho tornozelo (reflexos hipoativos)*	Addition

*Highlighted information represents what was modified or added to the original item.

Table 3 shows the results of the reliability analysis for the HINE total scores and for the five categories. We found strong internal consistency, and the range of Alpha if item deleted was 0.91 (0.90–0.91) for the 26 items. We also found strong interrater reliability for the total score (ICC_2.1_=0.95) and for the five categories.

**Table 3:** Reliability study results according to categories.

HINE scores	Interrater reliability ICC (95%CI)
Cranial nerve function	0.88 (0.79–0.94)
Posture	0.89 (0.80–0.94)
Movements	0.83 (0.69–0.91)
Tone	0.90 (0.82–0.95)
Reflexes and reactions	0.94 (0.88–0.97)
HINE total score	0.95 (0.90–0.97)

HINE: Hammersmith Infant Neurological Examination; ICC: intraclass correlation coefficient; CI: confidence interval.

## DISCUSSION

This study presents the process of the HINE-Br translation and the psychometric property evaluation. The translation process was successful and produced a version with minimal necessary changes from the original version. The HINE Portuguese-Brazil version presented strong reliability properties.

The HINE is a useful and valid tool for early detection of CP in infants around the world.^9^ Its website presents the Recording & Scoring Proforma translated into 15 different languages.^25^ Despite the several available HINE Recording & Scoring Proforma, few studies have described the translation process.^14,27^ The guidelines are important to ensure that, despite the necessary changes, the translation is faithful and similar to the original version of the instrument.^22,23^ The translation process of the HINE-Br followed the recommendations of internationally recognized guidelines,^22,23^ and was carried out by experienced professionals and by the developer of the instrument. A recent study described the same guidelines as the present study to translate the HINE into the Turkish language and also identified that the Turkish HINE version presented good psychometric properties.^27^

The reproducibility should be tested whenever an instrument undergoes an adaptation and/or translation process and when it is applied in different populations and contexts from those in which it was developed.^26^ Our results identified that the HINE-Br version presents appropriate internal consistency, and strong interrater reliability for the total scores and for the category scores. It should be noted that we did not find any other scientific articles in the literature that verified the internal consistency of the HINE items, thus, the present study contributes to the field by identifying that the items are homogeneous and measure the same construct.

The literature demonstrates that other translated versions of the HINE present good reliability rates. The HINE Turkish version was evaluated in 35 children by two physical therapists and presented strong interrater and intra-rater reliability (ICC>0.96).^27^ The study results also identified that the HINE scores were consistent with three different validity methods of predicting neurodevelopmental outcomes.^27^ A study conducted in Saudi Arabia^28^ evaluated the intra and interrater reliability in 31 children up to 12 months of age and found excellent results for the HINE global scores and its categories. The results of these studies corroborate the results of the present study and demonstrate that the HINE is a reliable instrument to assess the neuromotor development of children between 3 and 24 months of age. Despite being an easy-to-apply instrument, the good reliability rates found in the studies can be justified by the training carried out by the examiners and their experience in the pediatric area.

Our study followed the guidelines for methodological studies and included a representative sample of all ages covered by the assessment using the HINE. As a limitation, due to the pandemic period, we were unable to reach the sample size suggested by COSMIN for the reliability study (i.e., 50 participants). However, our results were significant, and the correlation found was similar to other studies.^27,28^ We also did not verify the other measure proprieties, such as intra-examiners, and we restricted a limited number of examiners, as the study took place during the COVID-19 pandemic. An investigation with greater variability among different health professionals could be important to reinforce the reliability of the instrument. The main objective of the HINE is to assist in the early diagnosis of CP and facilitate referrals for interventions. This is possible by identifying cutoff points that are valid for diagnosing whether or not the child is at high risk for CP. The literature already presents these cutoff points;^4,12^ however, future studies could verify the predictive validity properties of the HINE to detect CP in each age group of Brazilian children.

This study identified that the HINE-Br is a reliable instrument for the evaluation of Brazilian children in a public outpatient clinic, by professionals in the field of child health, such as pediatricians and physical therapists. The HINE is characterized by the literature as a useful tool in the detection of neuromotor alterations in infants at a high-risk of CP, and is possible to be applied in different environments, including hospitals, follow-up programs, and basic health units. However, in LMICs, few studies have reported evaluations for the early detection of CP using the HINE.^10,29^ Despite being one of the most prevalent neurological health conditions in childhood, there is a lack of epidemiological studies and guidelines on the early diagnosis of CP in Brazil.^30^ Thus, the current study makes available, to Brazilian health professionals, the translated Brazilian Portuguese version of a valid, low-cost, and easy-to-use instrument, recommended worldwide for the early detection of CP.

In conclusion, the HINE-Br presents adequate rates of internal consistency and reliability and can be used by pediatricians and physical therapists for the evaluation of children aged between 3 and 24 months at risk for CP.

## Data Availability

The database that originated the article is available with the corresponding author.
